# Radiotherapy for recurrent central Giant cell granuloma: a case report

**DOI:** 10.1186/s13014-019-1336-7

**Published:** 2019-07-19

**Authors:** Qi Zhang, Zelai He, Gengming Wang, Hao Jiang

**Affiliations:** 1 Department of Radiation Oncology, The First Affiliated Hospital of Bengbu Medical University and Tumor Hospital Affiliated to Bengbu Medical University, 287 Changhuai Road, Bengbu, Anhui 233004 People’s Republic of China; 2 Anhui Province Key Laboratory of Translational Cancer Research Affiliated to Bengbu Medical University, Bengbu, 233004 China

**Keywords:** Central giant cell granuloma, Radiotherapy, Surgical resection

## Abstract

**Background:**

Central giant cell granuloma (CGCG) is a rare, non-neoplastic, benign lesion that exhibits expansive and osteolytic biological behavior. CGCG treatment and management is challenging for clinicians.

**Case presentation:**

This report presents the treatment and management of recurrent, aggressive CGCG after surgical resection. After informed consent was obtained, the patient underwent radiotherapy. The lesion size was reduced significantly, with no evidence of recurrence or malignant transformation.

**Conclusions:**

This treatment experience indicates that radiotherapy can be used as a rescue treatment for complicated CGCG involving vital neurovascular structures of the cranial base.

## Background

Central giant cell granuloma (CGCG) is a rare, non-neoplastic, benign lesion that exhibits expansive and osteolytic biological behavior [1]. The lesion principally affects the mandible and maxilla and rarely affects the skull or small bones of the hands and feet [2, 3]. To the best of our knowledge, the present case is the first report of recurrent CGCG of the nasal cavity and sinuses and its successful treatment by intensity-modulated radiotherapy.

## Clinical presentation

**Fig. 1:**
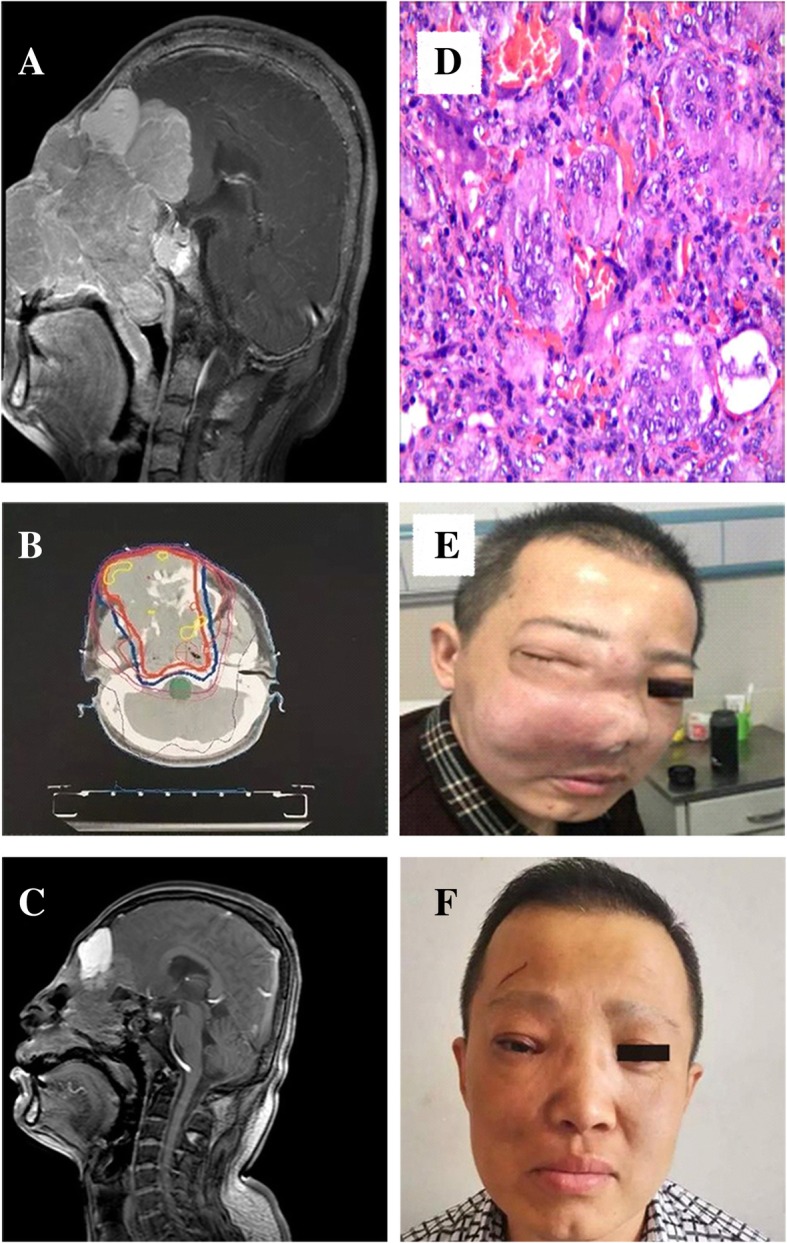
Clinical features of the patient. **a** Pathological features: hypertrophic shuttle fibroblasts scattered in the distribution of multicore giant cells; cells of different sizes; cells with small nuclei; no overtly mitotic cells; visible interstitial bleeding. **b** Pretreatment computed tomography (CT): the lesion involved in the right nasal cavity, maxillary sinus, right frontal lobe, right eye, and skull base bone. **c** Pretreatment MRI: isointense signals on T1-weighted imaging and more heterogeneous signals on T2-weighted imaging. The lesion primarily involved the nasal cavity, nasopharynx, bilateral ethmoid sinus, extraocular muscles, and frontal lobe of the parenchyma. Additionally, the bilateral ventricle was compressed. **d** Posttreatment MRI scan: right nasal cavity, maxillary sinus, ethmoid sinus and right side of the skin showed mixed T1 and T2 signals, and the edge was unclear; uneven signals showed enhanced abnormalities of the signal and unevenness near the maxillofacial bone; right frontal sinus expansion was observed, and the sinus cavity showed irregular cystic short T1 and slight T2 signals. The right frontal sinus showed an abnormal signal, which was considered a “mucous cyst”. **e** Pretreatment appearance. **f** Posttreatment appearance

The patient, a 35-year-old man without a previous history of trauma or predisposing factors, was initially referred to the Otorhinolaryngology Department in September 2014 for a neoplasm in the right nasal cavity. Surgical resection of the neoplasm was performed. The postoperative pathological findings supported the diagnosis of CGCG (Fig. 1d). After surgical resection, clinical and radiographic examinations revealed complete remission. The patient was referred to our center in December 2015 for massive facial swelling (Fig. 1e). The patient’s main complaint was “an intermittent headache lasting for more than three months, accompanied with a progressive decline in hearing lasting for one month”. Computed tomography (CT) revealed that the lesion affected the right nasal cavity, maxillary sinus, right frontal lobe, right eye, and skull base bone (Fig. 1b). Magnetic resonance imaging (MRI) showed a large lesion reaching 9.8 × 7.6 cm in size. The lesion primarily involved the nasal cavity, nasopharynx, and bilateral ethmoid sinus. Additionally, the invasive lesion expanded intracranially, affecting the eyes (bilateral), extraocular muscles, and frontal lobe of the parenchyma. Additionally, the bilateral ventricle was compressed (Fig. 1a). After informed consent was obtained, the patient underwent intensity-modulated radiotherapy with X-rays at 6 MV in 8 fields at a dose per fraction of 2 Gy and a total dose of 56 Gy. Fifteen months after the procedure, follow-up MRI was performed to reappraise the lesion. MRI showed almost complete remission (Fig. 1c and f). Furthermore, the patient experienced improvement in his neurological symptoms.

## Discussion

CGCG is a rare, non-neoplastic lesion. The World Health Organization classifies it as a type of benign lesion of bone, which always shows osteolytic biological behavior. The lesion was first reported by Jaffe in 1953 as a “giant cell repair granuloma” [4]. To date, the etiology of giant cell granuloma remains to be defined. Jaffe reported that it was associated with reparative mechanisms after trauma, while others questioned the “reparative” label and suggested that it should be avoided. Some scholars have suggested that it is not trauma related but is instead associated with the inflammatory response [5] or pregnancy [6]. Approximately 70% of CGCG lesions occur in the mandible. Reported cases outside the jaw include the orbital bone, temporal bone, ethmoid, saddle area, hand, short foot bones, and vertebrae.

Non-aggressive lesions do not perforate the cortical bone, and the recurrence rate is low. Aggressive lesions are characterized by rapid growth, perforation of the cortical bone, pain, tooth root resorption, anatomical destruction and dysfunction of involved organs, which results in a high recurrence rate [7, 8]. The primary sites in the present case were the nasal cavity and sinus, which have rarely been reported before. The recurrent lesion was large in volume and had a wide infiltration range. The patient suffered from headache and hearing loss, which were caused by the lesion invading the brain and cranial nerves. CGCG does not exhibit specific radiological features. Computed tomography (CT) plays an important role in determining the size and extent of the lesion and the presence of cortical expansion and bony destruction. It can be used as a tool for comparing the lesion before and after treatment [6]. Due to its low incidence, lack of specificity in terms of clinical manifestations, and nonspecific radiological features, this disease is likely to be clinically misdiagnosed. The clinical diagnosis of this disease should take into account the age at onset, clinical manifestations, radiological findings, pathology, and response to treatment.

CGCG can easily be confused with a tumor before surgery and is easily misdiagnosed as a bone giant cell tumor (GCT), as the histological differences between CGCG and GCT may be subtle. The pathological features of CGCG include multinucleated giant cells clustered around hemorrhagic foci, whereas the multinucleated giant cells of GCT tend to be densely packed and evenly distributed. The multinucleated giant cells of GCT are larger and may contain up to 50 nuclei, compared with the 10 to 15 nuclei typically seen in CGCG. New bone formation and collagen deposition are characteristics of CGCG but absent in GCT. The age at onset of CGCG is earlier than that of GCT, and CGCG most often involves the mandible or maxilla. The recurrence rate of CGCG ranges from 10 to 15%, and although this demonstrates aggressive local behavior, CGCG metastases have not been reported. GCT mostly involves the long bones and rarely involves the craniofacial region. In contrast to CGCG, GCT metastasis has been reported. The patient reported in this article did not have an abnormal calcium, alkaline phosphatase, or phosphate level [8].

At present, surgical curettage of the involved area or complete resection of the aggressive lesion are the most common therapies for CGCG. A study in the Netherlands showed that the progression-free survival rate was 93.2, 80.7, and 76.1% at 1, 3, and 5 years after surgery, respectively [8]. A series of studies have reported that the recurrence rate of the disease ranges from 11 to 49% and that recurrence mostly occurs within 2 years of surgery. Complete secondary resection, if possible, is beneficial for patients with recurrence. Pharmacological agents, including calcitonin [9, 10], IFN-α [8], and denosumab [11], are promising alternatives to surgical management. Pharmacological agents have the potential to decrease the recurrence rate, minimizing the morbidity associated with surgery, and even prevent the need for surgical intervention [9, 12, 13]. A double-blind clinical study reported that calcitonin significantly reduced the recurrence rate of central giant cell granuloma after curettage.

The recurrence rate in the experimental group and the control group was 9.1 and 53.8%, respectively. However, this drug treatment was only assessed based on a retrospective case analysis of a small population sample. Because of the benign characteristics and low incidence of CGCG, the use of radiotherapy for treating CGCG is rare. The role of adjuvant radiotherapy in eliminating residual giant cell tumor cells is controversial. It has been claimed that giant cell tumors are not radiosensitive and that radiation carries a significant risk of sarcomatous transformation. Adjuvant radiotherapy has only been suggested for patients who are not surgical candidates or are treated with partial resection or to minimize the risk of recurrence [14, 15]. For patients with extensive recurrence, the use of radiotherapy as the primary treatment has not yet been reported. To the best of our knowledge, the present case study is the first report of recurrent CGCG of the nasal cavity and sinuses successfully treated with fractionated radiotherapy. The selection of radiotherapy as an optimal treatment in this case was based on the consideration that the recurrent lesion was so large that complete resection could not be achieved. The patient had a wide range of lesion infiltration in the brain, nerve tissue, and eyeballs. Secondary surgery would have been difficult and would have caused serious organ dysfunction as a consequence of severe trauma to these vital organs. Furthermore, modern techniques for intensity-modulated radiotherapy are safe and effective. The reference radiotherapy dose is 40–56 Gy, according to related literature [15, 16]. The process of radiotherapy for this patient was divided into two stages. In stage 1, the patient underwent initial radiotherapy with a radiation dose of 40 Gy. Reappraisal of the lesion was performed by MRI, which showed that the lesion had diminished significantly. In stage 2, to improve the total dose delivered to the primary lesion location and to reduce the radiation dose delivered to other tissues, such as the brain stem and eyeballs, we designed a new radiotherapy dosage plan. The total radiotherapy dose delivered to the gross tumor volume (GTV) reached 56 Gy. At the 15-month follow-up visit, the lesion demonstrated a significant reduction in size, with no evidence of recurrence or malignant transformation. During radiotherapy, second-degree oral mucositis and third-degree keratitis occurred, from which the patient recovered after support treatment. No new late radiotherapy-related toxic side effects have occurred. The headache and hearing loss symptoms were also relieved. Continued long-term follow-up examinations are necessary to determine whether this lesion will continue to resolve or whether there is potential for malignant transformation.

## Conclusion

CGCG is a rare, benign lesion that exhibits expansive and osteolytic biological behavior. The diagnosis of this disease should take into consideration the patient’s age, clinical symptoms, radiological imaging findings, pathological features, and response to treatment. Although surgery is still the main treatment for CGCG, the results presented in this case study suggest that radiotherapy can be used as salvage treatment for complicated CGCG cases involving vital neurovascular structures of the cranial base.

## Data Availability

The datasets used and analyzed during the current study are available from the corresponding author on reasonable request.

## Abbreviations

CGCG: Central giant cell granuloma
CT: Computed tomography
GTV: Gross tumor volume
MRI: Magnetic resonance imaging
